# Reduced plasma TIA-1: bridging established pathology and novel biomarker potential

**DOI:** 10.1186/s13195-026-02020-9

**Published:** 2026-03-25

**Authors:** Neelam Younas, Abrar Younas, Peter Hermann, Leticia Camila Fernandez Flores, Kathrin Dittmar, Saima Zafar, Holger Budde, Tobias J. Legler, Tayyaba Saleem, Matthias Schmitz, Inga Zerr

**Affiliations:** 1 Department of Neurology, University Medical Center, National Reference Center for Surveillance of TSE, Robert-Koch-Strasse 40, Goettingen, 37075 Germany; 2 German Center for Neurodegenerative Diseases (DZNE), University of Goettingen, Von-Siebold-Straße 3A, Goettingen, Germany; 3 Department of Transfusion Medicine, University Medical Center Goettingen, Robert-Koch-Strasse 40, Goettingen, Germany

**Keywords:** TIA-1, Alzheimer’s disease, Plasma biomarker, Rapidly progressive AD, Neurodegenerative diseases

## Abstract

**Background:**

TIA-1 (T-cell intracellular antigen 1) is an RNA-binding protein central to the formation of stress granules (SGs) and modulation of inflammatory responses. Recent research has identified TIA-1 as a key player in neurodegenerative diseases, especially those involving Tau pathology such as in Alzheimer’s disease (AD), in a subtype-dependent manner. Disease-modifying therapies for AD and related dementias are emerging, but their effectiveness hinges on timely and accurate diagnosis. This underscores urgent need for easily accessible and reliable blood biomarkers for early detection, disease monitoring, and subtype differentiation.

**Methods:**

Accordingly, in this pilot study, we evaluated the plasma TIA-1 levels in healthy controls and across spectrum of neurodegenerative diseases to assess its clinical and biological significance. Plasma TIA-1 concentrations were measured in Alzheimer’s disease, mild cognitive impairment due to AD (MCI-AD), vascular cognitive impairment (VCI), and other neurodegenerative diseases (NDs: synucleinopathies and tauopathies), and compared to healthy controls. Receiver operating characteristic (ROC) analyses and correlation studies with established biomarkers and cognitive scores were performed.

**Results:**

Plasma TIA-1 levels were significantly reduced in AD, MCI-AD, VCI, and non-AD neurodegenerative diseases (synucleinopathies and tauopathies) compared to healthy controls, with the greatest reductions observed in non-AD neurodegenerative diseases. Strikingly, combined measurement of plasma TIA-1 and glial fibrillary acidic protein (GFAP) improved differentiation of AD subtypes, particularly distinguishing rpAD from non-rpAD cases. Plasma TIA-1 levels correlated with key biomarkers of neurodegeneration (p-Tau, Aβ42, NFL) and cognitive decline, especially in early AD (MCI-AD). Notably, plasma TIA-1 mirrored the discriminatory patterns of CSF t-Tau and p-Tau, distinguishing non-AD neurodegenerative diseases from specifically rpAD subtype, indicating that plasma TIA-1 captures the same underlying pathophysiological processes as the “gold standard” CSF markers.

**Conclusions:**

Plasma TIA-1 is a promising biomarker candidate for neurodegenerative diseases, with alterations that reflect disease presence and severity. In this pilot cohort, its combination with GFAP noticeably improved discrimination between AD subtypes, supporting its potential added value in composite biomarker approaches. Given its accessibility, TIA-1 is a strong candidate for further evaluation as part of future blood-based biomarker panels, although it’s clinical utility still needs confirmation in larger, independent studies.

**Supplementary Information:**

The online version contains supplementary material available at 10.1186/s13195-026-02020-9.

## BAckground

TIA-1 (T-cell intracellular antigen 1) is an RNA-binding protein central to the formation of stress granules [1], regulation of RNA metabolism [2, 3], and modulation of inflammatory responses [4, 5]. Recent research has identified TIA-1 as a key player in neurodegenerative diseases, especially those involving Tau pathology such as Alzheimer’s disease [5–7].

Alzheimer’s disease is the predominant form of dementia. Currently, 25% to 30% of AD patients are not correctly diagnosed at specified dementia units [8]. This is even more problematic at earlier phases of the disease, particularly in patients with a subjective cognitive decline or mild cognitive impairment (MCI) and without dementia [9]. Reliable biomarkers are needed for a correct diagnosis, but also for estimation of disease progression rate to enable selection of homogeneous cohorts in clinical trials.

Classically, Alzheimer’s disease is a slowly progressive form of dementia. However, multiple clinical phenotypes and progression variants have been reported for AD population [10, 11]. One extreme variant of AD spectrum is rapidly progressive AD (rpAD), which is associated with a very rapid cognitive decline and/or a reduced survival time [12–16]. Currently, there is no valid definition of rpAD [10, 11]. Based on the criteria used for the classification of rpAD, 10% to 30% of AD cases are characterized as rpAD [12]. The lack of distinct biomarkers and fast progression of the disease are the major challenges to distinguish it from other rapidly progressive dementias [17, 18] and for clinical management and research studies of rpAD cases.

Furthermore, pathological investigations highlight that the neurological diseases have a preclinical prodromal phase without any symptoms or very mild (non-specific) symptoms in spite of active pathological processes [9]. The identification of these underlying neuropathological processes is important for timely diagnosis and differentiation of AD dementia from other neurological disorders.

Recent evidence indicate a novel pathological feature of Tau protein in relation to RNA-binding proteins like TIA-1 [19]. Younas et al., [7] discovered a dysregulation of TIA-1 in human brain frontal cortex in Alzheimer’s disease, in a disease subtype-dependent manner. TIA-1 cytotoxic granule associated protein regulates multiple features of RNA metabolism but is best understood for its role in stress granule (SG) formation during stress response [20]. As a core marker of SGs, TIA-1 regulates pathophysiology (translocation, assembly and misfolding) of Tau protein in part through recruitment of phosphorylated-Tau oligomers into pathological stress granules [6, 19, 21]. In contrast, Tau affects TIA-1 distribution, increases SG formation and disturbs the standard clearance mechanisms of SGs. Different species of Tau, its localizations and disease stages can affect the interaction of Tau with SGs [19] which promotes axon-terminal loss, apoptosis and neurodegeneration specifically at advanced stages of the disease [5, 6, 19]. SGs and TIA-1 also contribute to RNA splicing defects, proliferation of microglia and phagocytosis, which contributes to disease progression [5].

An easily accessible and reliable biomarker directly linked with the pathophysiology of hallmark proteins of AD [6, 19], and neuroinflammation [5, 22] could be an important addition to the AD biomarker diagnostic toolkit, for example to monitor pharmacodynamics effects of novel therapies on neuroinflammation and neurodegeneration. In this pilot study, we evaluated the levels of TIA-1 in blood plasma of healthy controls and across spectrum of neurodegenerative diseases to assess its clinical and biological significance.

## Methods

### Study cohort and CSF biomarker profile

The plasma samples of Alzheimer’s disease and related neurodegenerative disorders (*n* = 178) were obtained from the Dementia Outpatient Clinic of the University of Medicine Goettingen and the German National CJD Surveillance Center. The healthy control samples (*n* = 57) were obtained from the Department of Transfusion Medicine of the University Medical Center Goettingen, Germany (approval no. 2/7/21). AD was diagnosed clinically using the National Institute on Aging–Alzheimer’s Association (NIA–AA) criteria [23] and in addition, defined by result of one or more positive CSF biomarkers of amyloid-pathology (A) or AD-related Tau-pathology (T) [24]. CSF p-Tau181, Aꞵ42, and Aꞵ40 were measured using ELISA kits from Fujirebio (Fujirebio, Ghent, Belgium). The established lab-specific cut-offs indicated pathological levels of p-Tau 181 > 60 pg/ml (T), Aꞵ42 < 450 pg/ml or Aꞵ ratio [(Aꞵ42/Aꞵ40)*10)] < 0.05 (A). Because CSF Aβ40 measurements were affected by methodological changes after 2010, these values ​​were normalized as previously described [11]. The diagnosis of MCI due to AD (MCI‑AD) required an amnestic MCI syndrome together with the same CSF AD biomarker profile as outlined above.

Vascular cognitive impairment was diagnosed following the recommendations of the Vascular Impairment of Cognition Classification Consensus Study [25], based on comprehensive clinical evaluation (including CSF analysis) that did not reveal evidence for non‑vascular brain pathology. Other neurodegenerative conditions, such as synucleinopathies and tauopathies, were classified using established clinical consensus criteria as reported earlier [26].

For the classification of rpAD and non-rpAD cases, the definitions of rapidly progressive AD dementia by Schmidt et al. 2011 [12] and Soto et al. 2008 [27] were used. All the patients with a loss of ≥ 6 points on the MMSE score within a duration of 12 months or less were categorized as rpAD. All the slowly progressing (non-rpAD) cases had a drop of < 6 points within one year of observation. Informed consent was acquired from all the participants of the study or their caregivers. The institutional review board (IRB) of the ethical committee from the University of Medicine Goettingen, Germany, approved all the procedures.

Biomarker analysis for the current study comprised the measurement of cerebrospinal fluid (CSF) and plasma proteins, total-Tau (t-Tau), phosphorylated tau (p-Tau181) and amyloid-beta (Aβ40, and Aβ42) [11]. Additionally, plasma levels of NFL, GFAP and LCN2 were also measured. For measurement of these proteins, ELISA kits (FUJIREBIO, Ghent Belgium) were used. Additionally, the ApoE genotype of the AD subtypes was also performed and added to the analyses. The ApoE genotype was performed using DNA strip technology (GenoType APOE, Bruker) (Supplementary Fig. 4B).

### Blood collection and measurement

For blood collection EDTA tubes were used. After collection, blood was centrifuged at 2000 xg for 10 min, and the plasma was separated and aliquoted in polypropylene tubes, and stored at -80 °C until further use as per recommendations of the international consensus. TIA-1 levels were measured using enzyme-Linked Immunosorbent Assay (ELISA) kits from Biobool (Catalog No. E027072) according to manufacturer’s instructions, including the use of recommended controls and calibration procedures. All samples were measured in duplicates on the same plate to minimize analytical variability. Although intra- and inter-assay coefficients of variation were not systematically documented, all measurements complied with the manufacturer’s quality control guidelines, ensuring reliable and reproducible results.

### Statistical analysis

Statistical testings were performed using GraphPad Prism (10. 01. 02) and *R* (4.1.0). Based on a Shapiro-Wilk test all the data were skewed, so nonparametric tests were applied. A Kruskal-Wallis test was used to compare data from three groups. Mann-Whitney tests were carried out to compare two groups. For correlation analysis, we applied Spearman’s ρ correlations. Additional analyses (covariate, linear mixed model) were also performed. Significance was considered for *p-*values < 0.05. Receiver operating characteristic curves (ROC) were obtained for TIA-1 levels to find out its diagnostic value. AUC (area under the curve) and a 95% confidence interval (CI) was calculated with GraphPad Prism (10.01.02). For cognitive and biomarker variables, data completeness was high. MMSE scores were missing for three participants and CSF biomarkers for one participant. Analyses involving MMSE or CSF biomarkers were performed using complete-case analysis, such that only participants with observed values for the respective variable were included; no imputation was performed.

This study was designed as an exploratory, hypothesis-generating pilot investigation. In light of the modest sample size, we did not apply a global correction for multiple testing (e.g., Bonferroni or false discovery rate [FDR]) in order to retain sensitivity to potentially meaningful effects. The results are therefore best viewed with a focus on effect sizes, the consistency of patterns across related analyses, and biological plausibility rather than on isolated p-values alone. These encouraging exploratory findings provide a basis for targeted confirmation in independent, adequately powered validation cohorts.

## Results

### Baseline characteristics of study subjects

In the current pilot study, a total of 178 participants were analyzed. The cohort comprised 70 individuals with Alzheimer’s disease cases (age 69.29 ± 9.45), 57 healthy controls (age: 60.12 *±* 6.42), 12 MCI-AD (67.83 ± 7.45), 17 VCI (69.24 *±* 12.88) and 22 neurodegenerative controls (ND: synucleinopathies and tauopathies) (68.23 *±* 7.9). All patients were enrolled from a prospective study on Alzheimer’s dementia.

The AD cases were divided into rapid progressors (rpAD) and non-rapid progressors (non-rpAD) according to the criteria of Schmidt et al. 2011 [12] and Soto et al. 2008 [27]. There were 45 non-rpAD (age: 70.80 ± 8.95) and 25 patients with rapidly progressive AD (age: 66.58 ± 9.91). Table 1 shows the baseline demographic characteristics, CSF biomarkers (Tau and Aꞵ) and plasma TIA-1 concentrations of the study participants. Notably, the CSF total-Tau (t-Tau) and phosphorylated-Tau (p-Tau) were significantly reduced in VCI- and ND-groups in comparison with rpAD cases (Supplementary Fig. 1B & C). The regression analysis with gender and age as covariates excluded the potential confounding effects of age and sex on the differences in plasma TIA-1 levels between groups (Supplementary Fig. 2B & C).

Additionally, CSF Aß40 concentrations were significantly higher in non-rpAD compared to rpAD cases (p-value = 0.0090; supplementary Fig. 1A and 2A). Comprehensive biomarker profiling was performed in blood plasma including measurement of total-Tau, p-Tau, Aꞵ40, Aꞵ42, GFAP, NFL, and LCN2 (Supplementary Table 1).

**Table 1:** Demographics of the study subjects (*n* = 178)

Cohort	*n*	Age (mean ± SD)	Sex (m/f)	MMSE	CSF (mean ± SD)				Plasma (mean ± SD)
	178		92/86		t-Tau (pg/mL)	*p*-Tau (pg/mL)	Aß1–40 (pg/mL)	Aß1–42 (pg/mL)	TIA-1 (pg/mL)
AD	70	69.29 ± 9.45	30/40	20.63 ± 5.962	760.1 ± 510.0	102.8 ± 49.51	16,389 ± 9104	779.1 ± 1148	1036 ± 658.6
Non-rpAD	45	70.80 ± 8.95	20/25	21.40 ± 6.084	738.6 ± 483.5	98.11 ± 36.78	18,277 ± 9349	769.6 ± 1242	946.3 ± 712.9
rpAD	25	66.58 ± 9.91	10/15	19.25 ± 5.589	740.3 ± 491.2	111.2 ± 66.73	12,851 ± 7602	796.2 ± 981.2	1197 ± 522.9
MCI-AD	12	67.83 ± 7.45	7/5	21.55 ± 4.321	750.2 ± 514.4	116.6 ± 59.82	15,493 ± 7117	591.2 ± 318.8	1199 ± 780.0
VCI	17	69.24 ± 12.88	10/7	25.71 ± 3.424	164.5 ± 60.12	41.29 ± 10.88	9998 ± 4630	1410 ± 1370	664.4 ± 299.8
ND	22	68.23 ± 7.9	16/6	NA	243.7 ± 115.1	39.59 ± 13.29	13,851 ± 4209	1117 ± 314.2	580.7 ± 346.0
HC	57	60.12 ± 6.42	21/20	NA	NA	NA	NA	NA	1943 ± 1304

*AD *Alzheimer’s disease, *rpAD *rapidly progressive AD, *MCI-AD* mild cognitive impairment-AD, *VCI *vascular cognitive impairment, *ND *neurodegenerative diseases (synucleinopathies and tauopathies), *HC *healthy controls, *SD* standard deviation, *MMSE *Mini-Mental Score Examination, *CSF *Cerebrospinal fluid, *TIA*-*1 *T-cell intracellular antigen 1

### Reduced levels of plasma TIA-1 across diagnostic groups

**Fig. 1:**
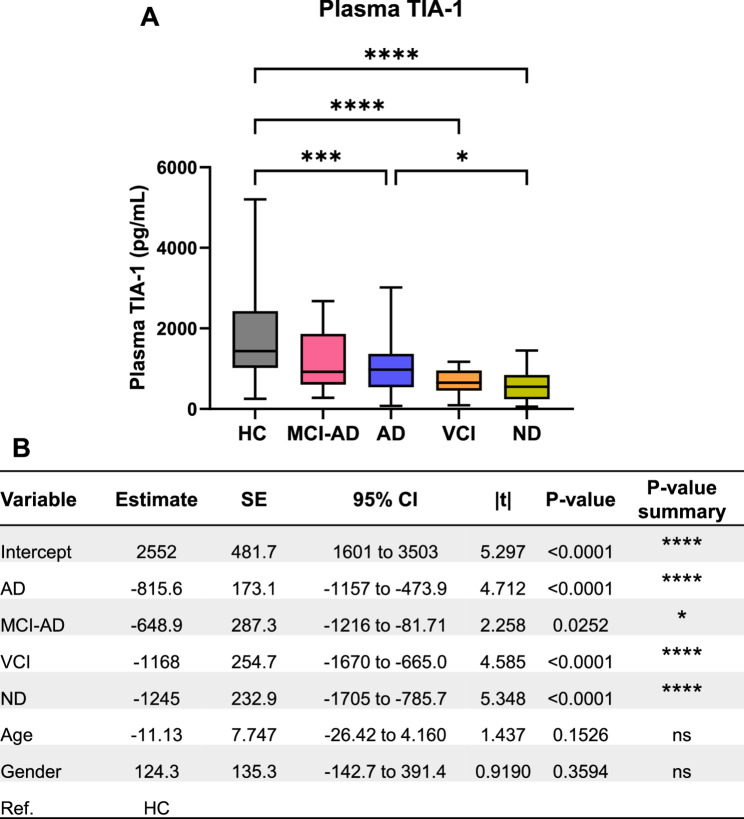
Plasma TIA-1 concentrations in healthy controls and neurodegenerative diseases. **A** Summary of the concentrations of plasma TIA-1 in the healthy controls (n = 57), AD (n = 70), VCI (n = 17), MCI-AD (n = 12) and ND (n = 22) groups. Data is shown as Mean ± SEM (Kruskal-Wallis test, *p-value < 0.05, **p-value < 0.01, ***p-value < 0.001, ****p-value < 0.0001). **B** Regression analysis including age and gender as covariates. Ref: Reference, HC: Healthy controls. AD: Alzheimer’s disease, MCI-AD: mild cognitive impairment-AD, VCI: vascular cognitive impairment, NDs: neurodegenerative diseases (synucleinopathies + tauopathies), HC: healthy controls, SE: standard error, Ref.: reference, CI: confidence interval

Initially, we performed a direct comparison between levels of TIA-1 in healthy controls (*n* = 57) and all disease groups including AD cases (*n* = 70), VCI (*n* = 17), MCI-AD (*n* = 12) and other neurodegenerative diseases (NDs: synucleinopathies and tauopathies, *n* = 22). Plasma TIA-1 levels were significantly reduced in AD cases (Estimate: −815.6; *p* < 0.0001) as compared with healthy controls. This reduction was more pronounced in VCI (Estimate: −1168; *p* < 0.0001) and ND groups (Estimate: −1245; *p* < 0.0001), when compared with healthy controls (Fig. 1A & B).

There was also a significant decrease in TIA-1 levels in ND group (*p* = 0.0329), in comparison with AD cases. These findings were robust in kruskall-Wallis test (Fig. 1A) and persisted after adjusting for age and gender, which were not significant predictors of TIA-1 levels (Fig. 1B). Interestingly, after adjusting for age and gender, we observed a significant reduction in TIA-1 levels in MCI-AD cases (Estimate: −648.9; *p* = 0.0252), as compared with healthy controls (Fig. 1B).

**Fig. 2:**
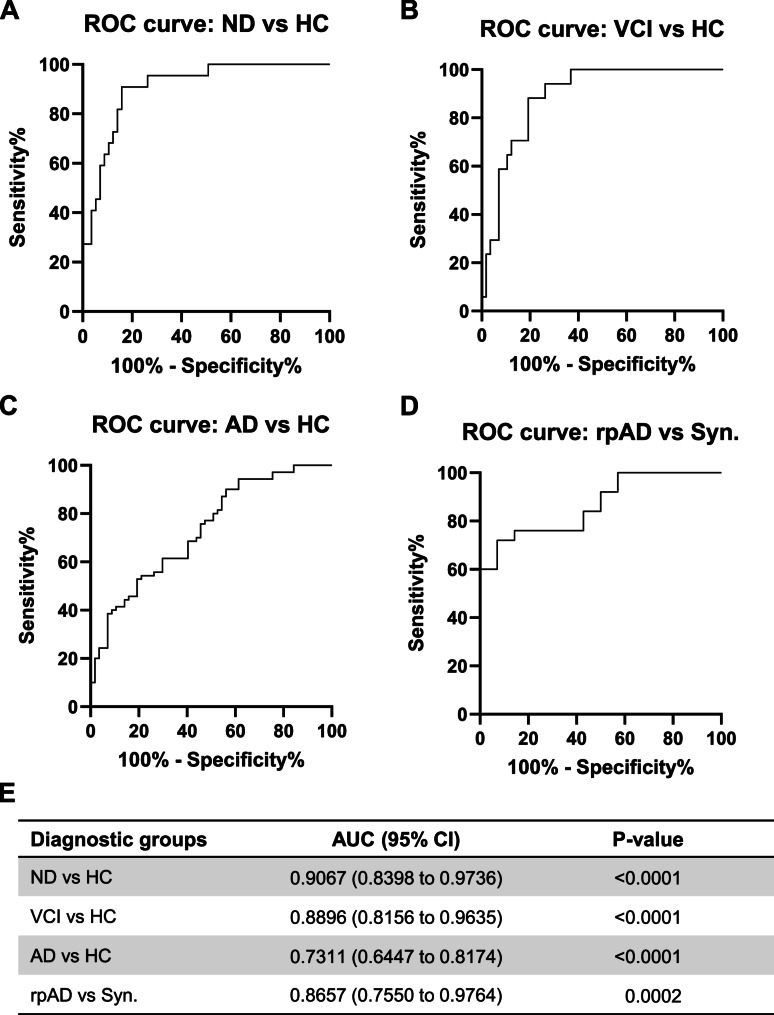
Receiver Operating Characteristic (ROC) analyses. **A-C** ROC curve between healthy controls (HC; *n* = 57) and NDs (neurodegenerative diseases; *n* = 22), VCI (vascular cognitive impairment; *n* = 17) and AD (Alzheimer’s disease; *n* = 70). **D **Roc curve analysis between neurodegenerative controls (NDs, *n* = 22) and rapidly progressive AD subtype (*n* = 25). **E** Area under the ROC curve and confidence interval for all analyses. NDs: neurodegenerative diseases (synucleinopathies and tauopathies), Syn. synucleinopathies

To assess the diagnostic value of plasma TIA-1, Receiver Operating Characteristic (ROC) analyses were performed. ROC analyses demonstrated excellent discrimination between disease groups and healthy controls. TIA-1 concentrations exhibited very good discriminating power for ND cases (AUC = 0.91, p-value < 0.0001) and VCI group (AUC = 0.8896, p-value < 0.0001), when compared with healthy controls (Fig. 2A, B & E). An AUC above 0.90 for ND group suggests that plasma TIA-1 can accurately distinguish neurodegenerative diseases (including synucleinopathies and tauopathies) from healthy individuals with high confidence. TIA-1 also showed moderate power to distinguish AD cases from healthy controls (AUC = 0.73, p-value < 0.0001) (Fig. 2C & E). The plasma TIA-1 levels can distinguish rapidly progressive AD (rpAD) from synucleinopathies with very good accuracy (AUC = 0.8657, p-value = 0.0002) (Fig. 2D & E).

**Fig. 3:**
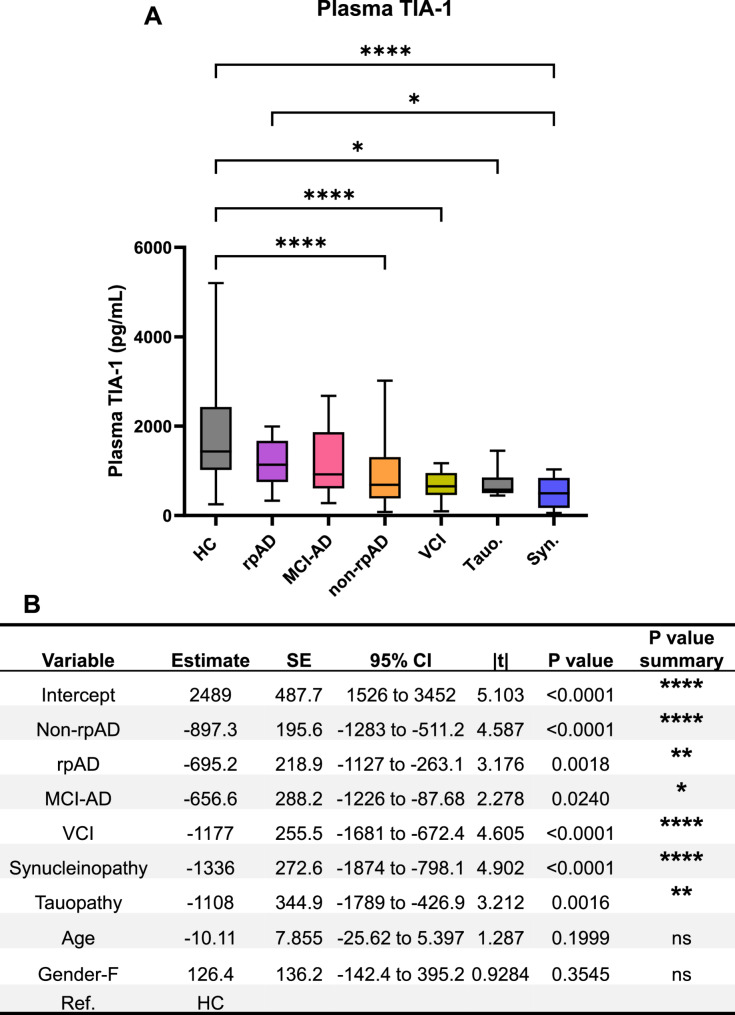
Plasma TIA-1 concentrations in all groups. **A** Summary of the concentrations of plasma TIA-1 in the healthy controls (*n* = 57), non-rpAD (*n* = 45), rpAD (*n* = 25), VCI (*n* = 17), MCI-AD (*n* = 12), synucleinopathies (*n* = 14) and tauopathies (*n* = 8). Data is shown as Mean ± SEM (Kruskal-Wallis test, *p-value < 0.05, **p-value < 0.01, ***p-value < 0.001, ****p-value < 0.0001). **B** Regression analysis including age and gender as covariates. Ref: Reference, HC: Healthy controls, rpAD: rapidly progressive AD, MCI-AD: mild cognitive impairment-AD, VCI: vascular cognitive impairment, Syn.: Synucleinopathies, Tauo.: Tauopathies

To find out differences in the fast and slow progressors, we separated AD cases into two subtypes, rapidly progressive AD (rpAD) and non-rpAD groups. Furthermore, we also segregated synucleinopathy and tauopathy cases due to strong connection between TIA-1 and Tau pathology [6, 7]. Both non-rpAD and rapidly progressive AD cases showed significant reductions in TIA-1 levels compared to healthy controls, with rpAD (p-value = 0.0018) showing a somewhat lesser reduction than non-rpAD (p-value < 0.0001) cases (Fig. 3A & B). Similarly, plasma TIA-1 levels were reduced in both tauopathy (p-value = 0.0016) and to a greater extent in synucleinopathy cases (p-value < 0.0001) compared with healthy controls (Fig. 3A & B).

Overall, plasma TIA-1 concentrations demonstrated strong to moderate ability to distinguish between healthy controls and various neurodegenerative conditions, with the highest accuracy for non-AD neurodegenerative diseases (synucleinopathies and tauopathies) and VCI cases. This supports its potential as a clinically useful marker for diagnosis and disease differentiation.

### Plasma composite biomarkers to distinguish rapidly progressive Alzheimer’s disease from other cognitive disorders

To find out if TIA-1 could be useful for discrimination of rpAD from non-rpAD casesꟷa desirable goal for early identification of rapidly progressive AD patientsꟷwe evaluated the utility of plasma composite biomarkers. This analysis included rpAD, non-rpAD, MCI-AD and VCI groups, as additional plasma markers were measured in these groups.

**Fig. 4:**
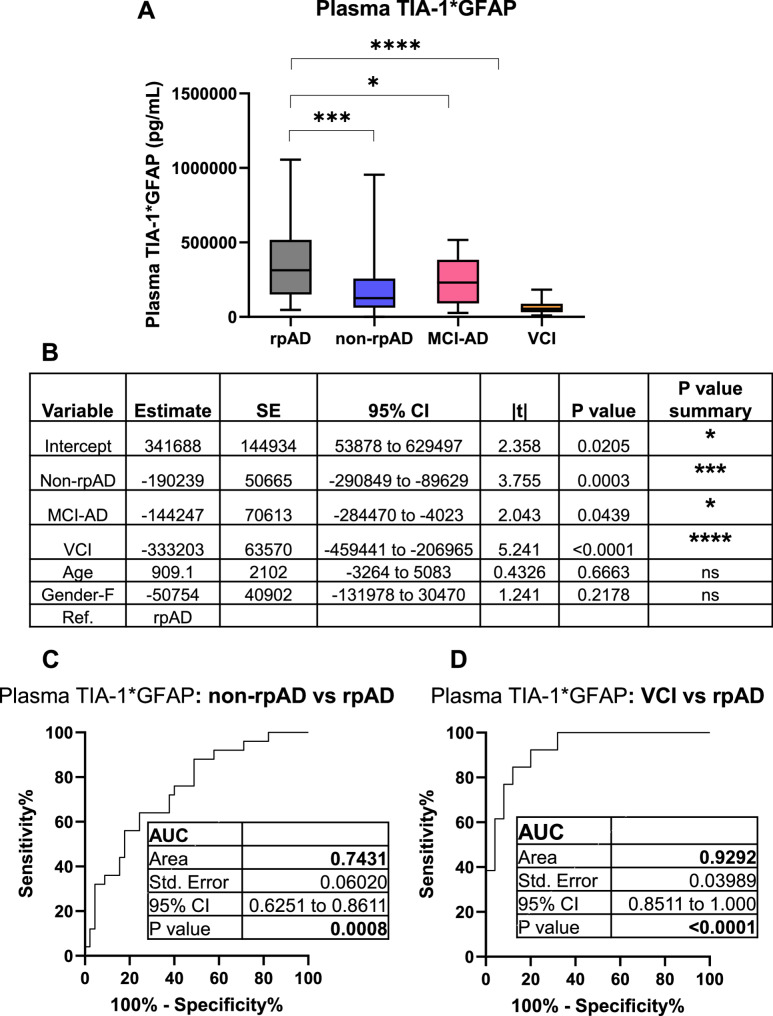
Intergroup comparison of plasma TIA-1 levels across neurodegenerative diseases. **A** The scatter plot is showing plasma TIA-1 levels (pg/mL) across rpAD (rapidly progressive variant of AD), non-rpAD (classical slowly progressive AD cases), MCI-AD (MCI due to AD) and VCI (vascular cognitive impairment). Data is shown as Mean ± SEM, *p-value < 0.05, **p-value < 0.01, ***p-value < 0.001). P-values are representative of regression analysis results. **B** Regression analysis including age and gender as covariates. Ref. Reference. **C & D** Receiver operating curve analysis between VCI and rpAD, and between non-rpAD and rpAD groups respectively

Notably, the combination of plasma TIA-1 with plasma GFAP significantly distinguished rpAD from non-rpAD cases (Estimate: -190239, *p*-value = 0.0003) (Fig. 4A, B & C) outperforming GFAP alone (Estimate: -94.18, *p*-value = 0.0018) (Supplementary Fig. 3A & B). The plasma TIA-1*GFAP levels were significantly reduced in MCI-AD cases (Estimate: -144247, *p*-value = 0.0439) as well, in comparison with rpAD (Fig. 4A & B). The VCI group showed greatest reduction in TIA-1*GFAP levels (Estimate: -333203, p-value < 0.0001) in comparison with rpAD cases (Fig. 4A & B). Age and gender were included in the model to exclude their confounding effects. These findings suggest that the combined plasma TIA-1*GFAP biomarker robustly distinguishes rpAD from other cognitive disorders, with the most pronounced reduction was observed in VCI group (AUC = 0.9292, *p* < 0.0001) (Fig. 4D).

Additionally, the ratio of plasma TIA-1/p-Tau was also significantly reduced in non-rpAD cases (Estimate: -897.7, *p* = 0.0155) in comparison with rpAD (Supplementary Fig. 3D). The ratio of TIA-1/Aꞵ40 was significantly higher in MCI-AD cases (Estimate: 52.80, *p* = 0.0387), in comparison with rpAD cases (Supplementary Fig. 3C). No significant differences in other plasma biomarkers (Tau, p-Tau, Aβ40, Aβ42, NFL, and LCN2) were detected among the various neurodegenerative disease groups.

Finally, when we compared plasma TIA-1 with traditional CSF markers (Tau and Aꞵ), Plasma TIA-1 showed exactly similar discriminatory patterns (distinguishing VCI and ND from rpAD) (Supplementary Fig. 1D & 2D) as observed with CSF biomarkers, although the CSF-Tau and p-Tau have stronger statistical power. 

### TIA-1 levels are reduced in individuals with isolated amyloid pathology (AT +/−) or neither pathology (AT −/−)

**Fig. 5:**
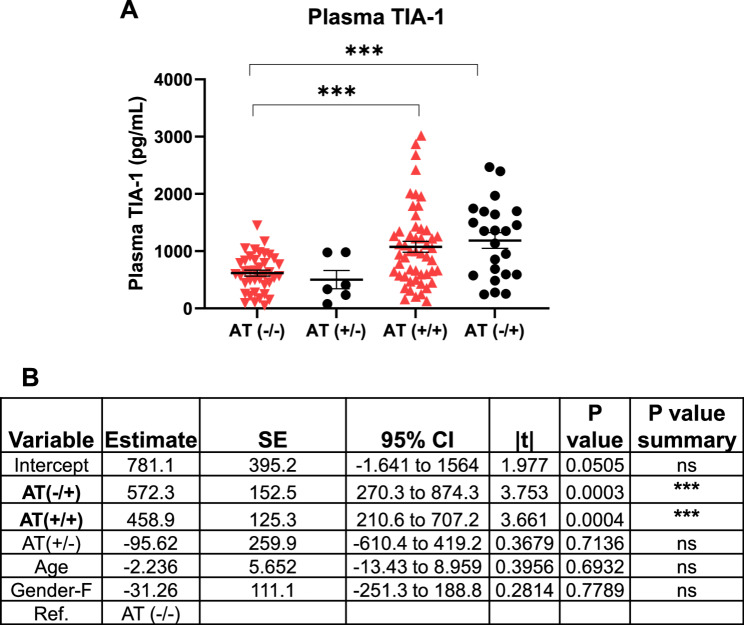
Plasma TIA-1 levels across four groups defined by AT (amyloid and Tau pathology) status. **A** The scatter plot is showing plasma TIA-1 levels (pg/mL) across AT (-/+): negative for amyloid, positive for Tau, AT (+/+): positive for both amyloid and Tau pathology, AT (-/-): negative for both amyloid and Tau, AT (+/-): positive for amyloid, negative for Tau. Data is shown as Mean ± SEM (*p-value < 0.05, **p-value < 0.01, ***p-value < 0.001), p-values are representative of regression analysis results. **B** Regression analysis including age and gender as covariates.SE: standard error, Gender-F: Female, Ref. Reference

Given a close interplay between TIA-1 and Tau pathology [5, 19], we sought to explore differences in the levels of TIA-1 in amyloid and Tau pathology (AT) continuum. Individuals with isolated Tau pathology AT (−/+) exhibited the highest plasma TIA-1 levels followed by individuals positive for both pathologies AT (+/+) (Fig. 5A). There was a significant difference (after adjusting for age and gender) in TIA-1 levels between AT (-/-) pathology cases, and individuals with isolated Tau pathology AT (-/+) (p-value = 0.0003), and between AT (-/-) and AT (+/+) cases (p-value = 0.0004). There were no significant differences observed between AT (-/+) and AT (+/+) (Fig. 5A & B). These results indicate that individuals with either isolated amyloid pathology (AT +/−) or neither pathology (AT −/−) have significantly lower plasma TIA-1 compared to the individuals having Tau pathology with or without amyloid pathology.

### Correlations

**Fig. 6:**
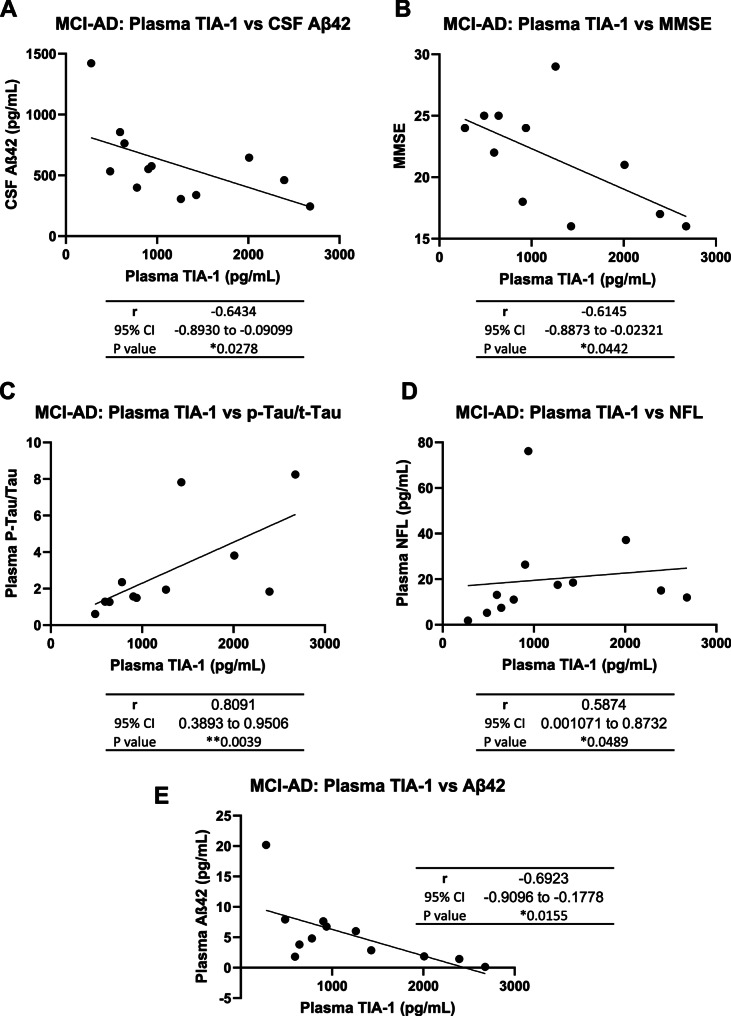
Correlation between plasma TIA-1 levels and traditional markers of neurodegeneration.** A-E** Spearman correlation between TIA-1 levels and CSF Aꞵ42 (**A**), MMSE score (**B**), plasma p-Tau/t-Tau ratio (**C**), plasma NFL (**D**) and plasma Aꞵ42 (**E**) in MCI-AD cases (*n* = 12). MCI-AD: Mild cognitive impairment due to AD, MMSE: Mini-Mental State Examination Score, p-Tau: phosphorylated Tau, t-Tau: total-Tau, NFL: Neurofilament light chain, CI: confidence interval

Interestingly, in MCI-AD, a significant negative correlation was observed between plasma TIA-1 and CSF Aꞵ42 (*r* = -0.6434, *p-value = 0.0278), and between plasma TIA-1 and Aꞵ42 (*r* = -0.6923; *p-value = 0.0155) (Fig. 6A & E). Furthermore, higher TIA-1 levels were associated with lower MMSE scores (*r* = -0.6145; *p-value = 0.0442) (Fig. 6B) indicating worse cognitive function. In contrast, a strong positive correlation was observed between plasma TIA-1 and p-Tau/t-Tau (*r* = 0.8091; **p-value = 0.0039) (Fig. 6C) supporting a relationship between TIA-1 and Tau-related neurodegeneration. Higher TIA-1 levels were associated with higher NFL levels (*r* = 0.5874; *p-value = 0.0489) in MCI-AD (Fig. 6D).

In VCI group, a positive correlation was observed between plasma TIA-1 and CSF p-Tau/t-Tau (*r* = 0.5763; *p-value = 0.0171) (Supplementary Fig. 4A). Additionally, we examined plasma TIA-1 levels across different ApoE genotypes to determine any genotype-associated variation. No significant differences were observed between groups (Supplementary Fig. 4B), suggesting that TIA-1 expression is independent of ApoE genotype status.

To assess whether these associations extend beyond the prodromal stage, we next examined the relationship between plasma TIA‑1 and core biomarkers as well as cognition in clinically manifest AD dementia (Supplementary excel file and Supplementary Fig. 5A-E). In this group, plasma TIA‑1 showed only a weak positive correlation with CSF Aꞵ42 (*r* = 0.2490; *p-value = 0.0130), while no significant associations were detected with MMSE, plasma NFL, or plasma Aꞵ42, and only modest correlations with CSF p‑Tau/t‑Tau (*r* = 0.2633, *p-value = 0.0301) (Supplementary Fig. 5C). Together with the strong, bidirectional correlations observed in MCI‑AD, these findings support a stage‑dependent role of TIA‑1, where dynamic changes in plasma TIA‑1 are most tightly linked to amyloid, Tau-related pathology, and cognitive performance at the MCI‑AD stage, but become attenuated once dementia is clinically manifest.

## Discussion

Early identification of molecular signatures that precipitate the pathogenesis of Alzheimer’s disease (AD) and other neurodegenerative conditions will likely maximize preventive and treatment success [28]. In this pilot study, we empirically demonstrated a reduction in plasma TIA‑1 levels across AD, MCI‑AD, and non‑AD neurodegenerative diseases (VCI and NDs: synucleinopathies and tauopathies) compared to healthy controls, with the greatest reductions observed in non‑AD disorders. Receiver Operating Characteristic analyses further confirmed that plasma TIA‑1 discriminates disease groups from healthy controls.

Reduction of TIA-1 has previously been observed in the brains of AD patients [7], with varying degrees of dysregulation across AD subtypes. Notably, rapidly progressive AD (rpAD) cases exhibit a less pronounced reduction in TIA-1 [7]. Our results align with these findings at the plasma level. Both AD subtypes—rapidly progressive (rpAD) and non‑rpAD—showed significant decreases, with rpAD showing less marked reduction. Given that rpAD is associated with distinct and more pathogenic Tau conformers [16], the differential TIA‑1 pattern observed here may reflect subtype‑specific molecular mechanisms or divergent pathological trajectories. However, these interpretations remain associative and warrant further mechanistic validation.

Mechanistically, TIA-1 occupies a central position at the interface of stress granule biology, Tau pathology, and neuroinflammation [6, 19, 29]. Experimental studies in mouse models have shown that reductions of TIA‑1 at early stages of tauopathy can be neuroprotective [22], whereas decreases at later stages may exacerbate neuroinflammation [5, 19]. Moreover, TIA-1 appears to exert cell type–specific effects, with distinct roles in neurons, microglia, and peripheral macrophages [29]. These findings drawn from prior mechanistic studies, provide a conceptual framework for interpreting our plasma data but should be regarded as speculative in the current context.

The cellular origin of circulating TIA-1 remains incompletely understood and likely involves both central and peripheral sources. Within the central nervous system, neuronal and glial stress granule dynamics, cell injury, and possibly extracellular vesicle–mediated release may all contribute to extracellular TIA-1. In the periphery, immune cells such as macrophages [30] and lymphocytes express TIA-1 [31] and may modulate plasma concentrations in response to systemic inflammation or immune activation. Thus, the circulating pool of TIA-1 likely represents a composite signal from both CNS and peripheral compartments. This dual origin should be considered when interpreting its disease-related changes and warrants further studies using cell-type–specific and longitudinal approaches.

In our cohort, greater reductions in plasma TIA‑1 in VCI and ND relative to AD imply that this marker may reflect a broader neuronal stress response rather than AD‑specific pathology. In line with its central role in stress granule assembly and Tau‑related neuroinflammation [6, 22], we hypothesize that plasma TIA‑1 may index upstream stress granule/tau‑oligomer biology across different disease etiologies. Differences between diagnostic groups could additionally be influenced by distinct immune or inflammatory profiles, which merits further investigation. Moreover, the close parallel between the discriminatory pattern of plasma TIA-1 and that of CSF t-Tau and p-Tau—particularly for separating VCI and ND from rpAD—supports the notion that TIA-1 is associated with Tau-related and overall neurodegenerative burden.

Correlative analyses further highlighted a potential stage-dependent role of TIA-1. In early, prodromal stages such as MCI-AD, modestly lower TIA-1 may reflect a more efficient resolution of the stress response, limiting the formation of persistent stress granules and the accumulation of toxic Tau oligomers, which has been linked to preserved synapses and better memory performance in experimental models [6, 22]. In this context, individuals with relatively lower TIA-1 within the MCI-AD group may therefore show better cognition and lower Aβ42 burden, consistent with a protective association at this stage. By contrast, in clinically manifest AD dementia, chronic disease progression is associated with sustained Tau pathology, widespread co-localization of TIA-1 with neurofibrillary pathology [7], and a generalized reduction of TIA-1 in the blood plasma in our cohort, which likely reflects advanced neurodegeneration rather than a beneficial adaptation. Thus, our data fit with a stage-dependent model in which TIA-1–mediated stress granule biology and Tau oligomer toxicity have different net effects in early versus late disease stages.

It is important to emphasize that, given the cross-sectional design of this study, our findings demonstrate associations rather than causal or directional relationships. While the observed reductions in plasma TIA-1 are biologically consistent with a potential adaptive or protective response in early disease stages [29], alternative explanations must be considered. These include altered peripheral expression of TIA-1, sequestration in tissues or cellular compartments, differences in protein clearance or degradation rates, and systemic immune or inflammatory effects that may independently influence plasma TIA-1 levels. These potential mechanisms form testable hypotheses for future work.

Beyond its behaviour as a single marker, our data suggest that TIA-1 may add value when combined with established plasma biomarkers. In line with growing literature supporting composite biomarker approaches [32], combining plasma TIA‑1 with plasma GFAP (TIA‑1*GFAP) provided greater discriminatory power for separating rpAD from non‑rpAD than GFAP alone and displayed strong separation of VCI from rpAD. The rationale for using a product term rather than a simple average was to emphasize individuals in whom both stress granule/Tau oligomer–related processes (indexed by TIA-1) and astroglial activation (indexed by GFAP) are concurrently elevated, under the hypothesis that rapid progression arises when these partly independent pathways are simultaneously engaged. This strategy is analogous to interaction or product terms commonly used in biomarker and risk‑score models and should be regarded as exploratory. While TIA-1 alone did not outperform GFAP on conventional diagnostic metrics and is unlikely to replace it as a stand-alone marker of rapid progression, the composite findings indicate that TIA-1 captures complementary variance that may refine risk stratification for aggressive disease courses.

Importantly, plasma TIA-1 is not proposed as a replacement for high-performing, AD-specific plasma markers such as p-Tau217, but rather as a complementary marker capturing a distinct, upstream pathophysiological axis related to stress granule formation [7, 33], Tau oligomer generation [6], and cellular stress responses [1]. In our cohort, plasma TIA-1 was associated with cognition and Aβ/tau-related measures particularly at earlier disease stages and showed graded modulation across AD, VCI, and ND, consistent with convergent stress-granule– and inflammation-related mechanisms that are not fully encompassed by p-Tau, GFAP, or NFL. Thus, TIA-1 is best viewed as an adjunct biomarker that may enhance existing plasma panels by indexing stress-granule/Tau-oligomer biology, rather than as a solitary diagnostic marker.

### Limitations

Overall, our results support plasma TIA-1 as a biologically grounded and accessible biomarker candidate that reports on stress granule– and Tau oligomer–related mechanisms with broad relevance across neurodegenerative and vascular disorders. At the same time, several limitations warrant caution. The study is a single-centre pilot with modest sample sizes in some subgroups (MCI-AD, rpAD, VCI, tauopathies), limiting statistical power, precision of effect estimates, and generalizability of subgroup and correlation findings. Multiple comparisons were not globally adjusted in order to preserve sensitivity in this exploratory context. Consequently, the present results should be viewed as preliminary and hypothesis-generating. Future research in larger, independent, and ideally multicenter cohorts—integrating longitudinal sampling, multimodal biomarkers, and mechanistic experimental data—will be required to validate plasma TIA-1 as a biomarker, clarify its cellular sources and temporal dynamics, and define its potential role within multidimensional biomarker panels for disease stratification and monitoring.

## Conclusions

TIA‑1 protein discriminates neurodegenerative disorders from healthy controls with clear disease‑specific patterns, supporting its role as a biologically plausible biomarker candidate. Plasma TIA‑1 is easily accessible and shows promising associations with established markers and clinical measures, and its combination with other plasma markers (e.g. GFAP) may add value for subtype differentiation and disease characterization in future studies. Targeting TIA‑1–related pathways may also represent an interesting therapeutic avenue by modulating stress granules, tau pathology, and neuroinflammatory responses. Overall, these findings are encouraging and hypothesis‑generating, and larger, independent studies will be important to confirm the diagnostic performance, clinical utility, and therapeutic implications of TIA‑1.

## Supplementary Information

Supplementary Material 1.

Supplementary Material 2.

## Data Availability

All data generated or analyzed during this study are included in the main article and its supplementary information.

## Abbreviations

Aβ40: Amyloid-beta precursor protein 1-40
Aβ42: Amyloid-beta precursor protein 1-42
AD: Alzheimer's disease
APOE: Apolipoprotein E
AUC: Area under curve
CI: Confidence interval
ELISA: Enzyme-linked Immunosorbent Assay
f: Female
FDR: False discovery rate
GFAP: Glial fibrillary acidic protein
HC: Healthy Controls
IRB: Institutional Review Board
LCN2: Lipocalin 2
m: Male
MCI: Mild cognitive impairment
MCI-AD: Mild cognitive impairment due to AD
MMSE: Mini-Mental State Examination
mL: Milli litre
NDs: Neurodegenerative diseases
NFL: Neurofilament light chain
non-rpAD: Non-rapidly progressive Alzheimer’s disease
pg: picogram
p-Tau: Phosphorylated Tau Threonine 181
Ref: Reference
ROC: Receiver operating characteristic
rpAD: Rapidly progressive AD
SE: Standard error
SEM: Standard error of mean
SGs: Stress granules
Syn: Synucleinopathies
t-Tau: Total Tau
TIA-1: T-cell intracellular antigen 1
VCI: Vascular cognitive impairment
