# Supplementary material for: Reduced plasma TIA-1: bridging established pathology and novel biomarker potential
Source: Alzheimers Res Ther. 2026 Mar 25;18:78. doi: 10.1186/s13195-026-02020-9 (PMC13063679; doi:10.1186/s13195-026-02020-9)
Supplement: Supplementary file 1 — Supplementary Material 1. [file 13195_2026_2020_MOESM1_ESM.docx]

**Reduced plasma TIA-1: Bridging established pathology and novel biomarker potential**

Neelam Younas*^#1,2^, Abrar Younas^#1,2^, Peter Hermann^#1^, Leticia Camila Flores Fernandez^1^, Kathrin Dittmar^1^, Saima Zafar^1^, Holger Budde^3^, Tobias J. Legler^3^, Tayyaba Saleem^1,2^, Matthias Schmitz^1,2^, Inga Zerr^1,2^

**^1^** University Medical Center Goettingen, National Reference Center for Surveillance of TSE, Department of Neurology, Robert-Koch-Strasse 40, Goettingen, Germany.

**^2^**University of Goettingen, German Center for Neurodegenerative Diseases (DZNE), Von-Siebold-Straße 3A, Goettingen, Germany.

**^3^**University Medical Center Goettingen, Department of Transfusion Medicine, Robert-Koch-Strasse 40, Goettingen, Germany

**#** Equal contribution

*Correspondence to Dr. Neelam Younas

Prion Research Group

National Reference Center for Surveillance of TSE

Department of Neurology, University Medical Center

Robert-Koch-Strasse, 40, 37075 Goettingen, Germany

Tel: +49 (551) 39-65398

Email: neelam.younas@med.uni-goettingen.de

**Supplementary Table 1**: Demographics of the study subjects and plasma biomarkers (n=178).

| **Cohort** | **n** | **Age *(mean ± SD*)** | **Sex (m/f)** | **MMSE** | **Plasma (*mean ± SD*)** | | | | | | | **Plasma (*mean ± SD*)** |
| --- | --- | --- | --- | --- | --- | --- | --- | --- | --- | --- | --- | --- |
|  | 178 |  | 92/86 |  | **t-Tau** (pg/mL) | **p-Tau** (pg/mL) | **Aß1-40** (pg/mL) | **Aß1-42** (pg/mL) | **NFL** (pg/mL) | **GFAP**  (pg/mL) | **LCN2**  (ng/mL) | **TIA-1** (pg/mL) |
| AD | 70 | 69.29 ± 9.45 | 30/40 | 20.63 ± 5.962 | 1.557 ± 1.990 | 2.916 ± 3.674 | 113.2 ± 75.95 | 5.408 ± 3.991 | 24.13 ± 31.18 | 237.2 ± 129.9 | 47.87 ± 35.97 | 1036 ± 658.6 |
| Non-rpAD | 45 | 70.80 ± 8.95 | 20/25 | 21.40 ± 6.084 | 1.741 ± 2.365 | 2.353 ± 1.810 | 115.2 ± 74.29 | 5.651 ± 4.004 | 26.17 ± 38.70 | 205.0 ± 113.8 | 48.26  ± 37.72 | 946.3 ± 712.9 |
| rpAD | 25 | 66.58 ± 9.91 | 10/15 | 19.25 ± 5.589 | 1.221 ± 0.954 | 1.940 ± 1.387 | 109.5 ± 80.33 | 4.973 ± 4.015 | 20.56 ± 7.131 | 293.9 ± 139.1 | 47.16 ± 33.36 | 1197 ± 522.9 |
| MCI-AD | 12 | 67.83 ± 7.45 | 7/5 | 21.55 ± 4.321 | 1.376 ± 0.914 | 2.342 ± 1.444 | 84.83 ± 63.52 | 5.440 ± 5.313 | 20.11 ± 20.05 | 204.4 ± 110.3 | 45.68 ± 23.39 | 1199 ± 780.0 |
| VCI | 17 | 69.24 ± 12.88 | 10/7 | 25.71 ± 3.424 | 1.984 ± 1.033 | 0.9166 ± 0.353 | 167.8 ± 48.23 | 7.173 ± 2.049 | 24.42 ± 21.20 | 95.28 ± 54.98 | 41.78 ± 21.66 | 664.4 ± 299.8 |
| ND | 22 | 68.23 ± 7.9 | 16/6 | NA | NA | NA | NA | NA | NA | NA | NA | 580.7 ± 346.0 |
| HC | 57 | 60.12 ± 6.42 | 21/20 | NA | NA | NA | NA | NA | NA | NA | NA | 1943 ± 1304 |

AD: Alzheimer’s disease, rpAD: rapidly progressive AD, MCI-AD: mild cognitive impairment-AD, VCI: vascular cognitive impairment, ND: neurodegenerative controls (synucleinopathies and tauopathies), HC: healthy controls, SD: standard deviation, MMSE: Mini-Mental State Examination, CSF: Cerebrospinal fluid, m: male, f: female, t-Tau: total tau, p-Tau: phosphorylated-Tau181, Aꞵ: Amyloid beta, NFL: neurofilament light chain, GFAP: glial fibrillary acidic protein, LCN2: lipocalin 2, TIA-1: T-cell intracellular antigen-1.

**Suppl. Fig. 1**: **CSF biomarkers (Aꞵ and Tau) and plasma TIA-1 concentrations of the study participants**. **A-C)** The scatter plot is showing CSF biomarkers (Aꞵ40, t-Tau and p-Tau). **D)** plasma TIA-1 across rpAD, non-rpAD, MCI-AD, VCI and other neurodegenerative diseases (ND). Data is shown as Mean ± SEM, *p-value < 0.05, **p-value < 0.01, ***p-value < 0.001, ****p-value < 0.0001). P-values are representative of regression analysis results including age and gender as covariates.

**Suppl. Fig. 2: Regression analysis results of CSF biomarkers and plasma TIA-1 including age and gender as co-variates. A-D)** For CSF Aꞵ40, t-Tau, p-Tau and **D)** plasma TIA-1 across rpAD, non-rpAD, MCI-AD, VCI and ND group (including synucleinopathies and tauopathies). Ref. Reference. rpAD: rapidly progressive Alzheimer’s disease, MCI-AD: MCI due to AD, VCI: vascular cognitive impairment, NDs: neurodegenerative diseases (synucleinopathies and tauopathies). SE: standard error, CI: confidence interval, Ref.: Reference.

**Suppl. Fig. 3: Plasma biomarkers (GFAP, TIA-1/Aꞵ40, TIA-1/p-Tau) across diagnostic groups. A)** Concentrations of plasma GFAP in rpAD (n = 25), non-rpAD (45), MCI-AD (n = 12) and VCI (n = 17) groups. Data is shown as Mean ± SEM (Kruskal-Wallis test, *p-value < 0.05, **p-value < 0.01, ***p-value < 0.001, ****p-value < 0.0001). **B)** A regression model was conducted for GFAP including age and gender as covariates. **C & D**) Regression analysis outcome for TIA-1/Aꞵ40 and TIA-1/p-Tau with gender and age as covariates. Ref: Reference, HC: Healthy controls. SE: standard error, CI: confidence interval.

**Suppl. Fig. 4: Correlation between plasma TIA-1 levels and CSF biomarkers in VCI. A**) Spearman correlation between plasma TIA-1 levels and CSF p-Tau/t-Tau in VCI (vascular cognitive impairment) (n = 17). CI: confidence interval, t-Tau: total Tau, p-Tau: phospho-Tau181. **B)** TIA-1 concentrations across ApoE genotypes**,** E2/3 (n = 3), E3/3 (n = 23), E3/4 (n= 36), E4/4 (n= 8).

**Suppl. Fig. 5: Correlation between TIA-1, MMSE and core AD biomarkers. A-E)** Spearman correlation between TIA-1 levels and CSF Aꞵ42 (**A**), MMSE score (**B**), CSF p-Tau/t-Tau ratio (**C**), plasma NFL (**D**) and plasma Aꞵ42 (**E**) in AD cases (n = 68). AD: Alzheimer’s disease, MMSE: Mini-Mental Examination Score, P-Tau: phosphorylated Tau, t-Tau: total-Tau, NFL: Neurofilament light chain, CI: confidence interval.
